# In Vitro Hypoglycemic Activities of *Lactobacilli* and *Bifidobacterium* Strains from Healthy Children’s Sources and Their Effect on Stimulating GLP-1 Secretion in STC-1 Cells

**DOI:** 10.3390/foods13040519

**Published:** 2024-02-07

**Authors:** Zhiliang Cheng, Jingru Chen, Yulong Zhang, Xinyi Li, Ning Zhang, Fei Liu, Yuehua Jiao

**Affiliations:** 1 Key Laboratory of Dairy Science-Ministry of Education, Food College, Northeast Agricultural University, Harbin 150030, China; c15333638933@126.com (Z.C.); chenjingru121@163.com (J.C.); 15036726189@163.com (Y.Z.); 15145007118@163.com (X.L.); 15848981381@163.com (N.Z.); 2 Center of Drug Safety Evaluation, Heilongjiang University of Chinese Medicine, Harbin 150040, China

**Keywords:** *Lactobacilli* and *Bifidobacterium*, antioxidant activity, type II diabetes mellitus, STC-1 cells, GLP-1

## Abstract

A long-term use of chemical drugs cannot cure type II diabetes mellitus (T2DM) and their numerous toxic side effects can be harmful to human health. In recent years, probiotics have emerged as a natural resource to replace chemical drugs in alleviating many human ailments. Healthy children’s intestines have a lot of colonized *Lactobacilli* and *Bifidobacterium*, and these beneficial bacteria can help promote overall health. The objective of this study was to isolate potential antidiabetic probiotic strains from healthy children and evaluate their application prospects. Firstly, *Lactobacillus* and *Bifidobacterium* strains were isolated from healthy children’s feces and identified by the *pheS* or *clpC* genes with their respective 16S rRNA genes. Then, hydrophobicity, artificial gastrointestinal fluid tolerance, α-Glucosidase and Dipeptidyl peptidase IV (DPP-IV) inhibitory activities of isolated strains were determined, and antioxidant activities and promoting secretion of GLP-1 in STC-1 cells of candidate strains were tested. Results showed that 6 strains of *Lactobacillus* and *Bifidobacterium* were obtained from the feces of healthy children aged 3 years, respectively, including *Lacticaseibacillus paracasei* L-21 and L-25, *Levilactobacillus brevis* L-16, *Lentilactobacillus buchneri* L-9, *Lactiplantibacillus plantarum* L-8 and L-3, *Bifidobacterium bifidum* 11-1 and B-84, *Bifidobacterium longum* subsp. *longum* 6-1, 6-2, B42 and B53. The hydrophobicity and auto-aggregation levels of all these strains were higher than 30% and 50%, respectively, and the decrease in the number of colonies of all strains in the artificial gastrointestinal fluid was less than 2 log CFU/mL. Strains L-3, L-8, L-9, L-21, 6-1, 11-1, B53 and B84 were selected based on their high α-glucosidase inhibitory activity and DPP-IV inhibitory activity, and results of the antioxidant capacity assay showed that the remaining strains all had intense comprehensive antioxidant activity. Additionally, *Lacticaseibacillus paracasei* L-21 and *Bifidobacterium longum* subsp. *longum* B-53 had the most substantial prompting effect on GLP-1 secretion in the STC-1 cell line. These results indicated that *Lacticaseibacillus paracasei* L-21 and *Bifidobacterium longum* subsp. *longum* B-53 could be used as a potential antidiabetic strain; thus, its application as a food supplement and drug ingredient could be recommended after in vivo mitigation of type II diabetes test.

## 1. Introduction

In recent years, improving human living standards has greatly increased the number of patients with diabetes. It is expected that by 2040, the number of adult diabetes patients worldwide will exceed 600 million, of which type II diabetes mellitus (T2DM) will account for 90%. In the future, T2DM will be one of the biggest global health challenges [1,2]. Obesity caused by high-carbohydrate and high-fat diets is the key reason for the occurrence of T2DM [3]. After the human body consumes a high-fat and high-carbohydrate diet for a long time, the pancreatic β-cells are overloaded and destroyed. The pancreatic β-cells destroyed will cause the body’s organs and tissues to reduce insulin-related receptors, showing insensitivity to insulin gradually, that is, Insulin Resistance (IR) [4]. IR contributes to the inability to lower blood glucose levels, and hyperglycemia occurs. When the fasting blood sugar concentration in the human body exceeds 11.1 mmol/L, one will suffer from T2DM [5]. T2DM can also lead to many complications, such as Retinopathy [6], Periodontitis [7], non-alcoholic fatty liver disease [8], atherosclerosis [9], etc. It brings serious harm to human health.

The infant’s intestinal microbiota is mainly derived from its mother and is affected by the mode of delivery and infant feeding practices [10]. The high proportion of *Lactobacilli* in the amniotic fluid and vagina can maintain the low pH environment of the maternal birth canal, which results in a higher abundance of *Lactobacilli* in the intestinal tract of infants born through vaginal delivery [11]. Due to the intake of breast milk, strictly anaerobic bacteria such as *Bifidobacterium* begin to colonize the intestines around 1 week after birth, and their abundance reaches its peak before the baby is 1 year old. However, when children aged 1 to 3 years old consume new formula milk powder and other foods, the intestinal pH gradually decreases, which contributes to the colonization of anaerobic bacteria and symbiosis with facultative anaerobic bacteria, and the intestinal microbiota also tends to stabilize [12]. In summary, the feces of 3-year-old healthy children contain high levels of *Lactobacilli* and *Bifidobacteria* suitable for colonizing the host and exerting probiotic effects. Many studies have demonstrated that *Lactobacilli* and *Bifidobacteria* can stimulate intestinal L cells to secrete Glucagon-like Peptide-1 (GLP-1) [13]. GLP-1 is a peptide hormone composed of 37 amino acids. The amino acid sequence of GLP-1 consists of all 29 amino acid sequences of glucagon and 8 amino acid sequences located at the carbon terminus. Therefore, GLP-1 can compete with glucagon for the binding site of its related receptor, inhibiting the increase in blood glucose concentration [14]. In addition, GLP-1 can improve insulin sensitivity and inhibit pancreatic β-cells apoptosis, which is important for the alleviation of T2DM [15]. The STC-1 cells (intestinal secretin tumor cell line) possess many characteristics of native enteroendocrine cells, which are often used as a screening platform to identify substances that modulate gastrointestinal hormone secretion in vitro. Many studies have shown that STC-1 cells are suitable for in vitro screening of substances that promote the secretion of intestinal hormones such as GLP-1, Glucose-dependent Insulinotropic Polypeptide (GIP) and Cholecystokinin (CCK) [16,17,18]. Therefore, in this investigation, *Lactobacilli* and *Bifidobacterium* were used to act on STC-1 cells, and the mRNA expression levels of pro-glucagon and PCSK1 gene were measured to screen for dominant strains with solid effects on promoting GLP-1 secretion.

In addition to having the ability to adapt to the host’s internal environment and colonize the body, strains that can alleviate T2DM also should have high α-glucosidase inhibitory activity and Dipeptidyl peptidase IV (DPP-IV) inhibitory activity. α-glucosidase is a digestive enzyme that acts on the α-glucosidic bonds of disaccharides and oligosaccharides to release glucose. The released glucose passes through the digestive system and is eventually absorbed by the villi of the small intestine into the blood, causing blood sugar to rise [19]. However, T2DM patients have limited insulin secretion due to a damaged pancreas and it is challenging to lower blood sugar on their own. Substances with high α-glucosidase inhibitory activity can help T2DM patients lower blood sugar. Substances with vigorous DPP-IV inhibitory activity achieve hypoglycemia by inhibiting the effects of GLP-1 degradation, prolonging gastric emptying time and decreasing appetite in T2DM patients [20]. Furthermore, as the metabolic environment changes, probiotics will produce more reactive oxygen species while producing metabolites, causing oxidative stress to themselves and harming the host. Therefore, intense antioxidant activity also guarantees the probiotic properties of the bacteria [21].

Thus, the present study aims to screen potential strains that alleviate T2DM and evaluate the strains with more substantial effects on promoting the secretion of GLP-1 from STC-1 cells based on biological properties such as strain tolerance, hypoglycemic activity and antioxidant activity.

## 2. Materials and Methods

### 2.1. Chemicals and Reagents

Pepsin, trypsin, bile salt, Pyrogallol, L(+)-ascorbic acid, Ferrous sulfate, potassium ferricyanide and trichloroacetic acid were purchased from Solarbio Science & Technology Co., Ltd. (Beijing, China). DMEM medium and special modified DMEM medium were obtained from Dalian Meilun Biotech Co., Ltd. (Dalian, China). RNA Isolation Kit was purchased from Vazyme Biotech Co., Ltd. (Nanjing, China). Fluorescent quantitation and a Reverse Transcription kit were obtained from United States Biological (Shanghai, China). ELISA kit was purchased from Chenglin Biotechnology Co., Ltd. (Beijing, China). DPP-IV was purchased from Sigma Chemical (St. Louis, MO, USA). All other chemicals and reagents were purchased from Beyotime Biotechnology Co., Ltd. (Shanghai, China).

### 2.2. Sample Selection and Collection

Feces were collected from five 3-year-old healthy children in Harbin, they all have no gastrointestinal complaints and have not taken any medicine and probiotics in the past three months. All feces were transferred in sterile sampling tubes containing pre-reduced water solution (containing 3 g/L Soya Peptone and 3 g/L L-cysteine hydrochloride), sealed and stored in the ice box. Then, diluted spread on de Man, Rogosa and Sharpe (MRS) medium (Haibo, Qingdao, China) and modified MRS (with 0.05% cysteine hydrochloride added, mMRS) agar plate, respectively, within 2 h and incubated anaerobically at 37 °C.

### 2.3. Strains and Cell Culture

*Lacticaseibacillus rhamnosus* GG and *Bifidobacterium animalis* subsp. *lactis* BB12 strains served as the reference strain in this study and were stored in the Dairy Industry Culture Collection of the Key Laboratory of Dairy Science of the Northeast Agricultural University, Ministry of Education, China (DICC). *Lactobacilli* were incubated in MRS broth (with inoculum amount of 2%, *v*/*v*) at 37 °C for 24 h. *Bifidobacteria* were anaerobically incubated in mMRS broth (with inoculum amount of 2%, *v*/*v*) at 37 °C for 24 h. All strains are subcultured three times prior to use. STC-1 cell line (Shangcheng Beinachuanglian Biotechnology Co., Ltd., Xinyang, China) was cultured in DMEM medium containing 10% fetal calf serum in 5% CO_2_/95% air at 37 °C.

### 2.4. Isolation and Identification of Strains

#### 2.4.1. Isolation of Strains

Fecal samples were mixed with dilution solution at 1:9 and gradient diluted to 10^−5^, and then 100 μL of each sample was taken and spread on MRS and mMRS agar, incubated at 37 °C for 48~72 h. Different colonies on the plates were picked and purified with streaking three consecutive generations on an agar plate. Isolated strains were preserved in 15% glycerol at −80 °C.

#### 2.4.2. Bacterial Genomic DNA Extraction and Identification

The bacterial genomic DNA was extracted according to the instructions of the RNA Isolation Kit. Then, the 16S rRNA gene sequence of strains was amplified according to the instructions of the PCR amplification kit. According to the methods of Naser [22] and Ventura [23], the housekeeping genes *pheS* and *ClpC* of *Lactobacillus* and *Bifidobacterium* were selected to perform PCR amplification, respectively. All primer sequences are shown in Table 1.

#### 2.4.3. Phylogenetic Tree Construction

PCR amplification products were sequenced by BGI Genomics Co., Ltd. (Guangzhou, China), and the BLAST algorithm (http://blast.ncbi.nlm.nih.gov/Blast.cgi, accessed on 18 June 2023; National Center for Biotechnology Information, Bethesda, MD, USA) was used to search strains with already known taxonomic status, which has the highest 16S rDNA sequence similarity with isolated strains. Then, 16S rDNA sequences of the corresponding type strains in the GenBank were extracted, and a phylogenetic tree was constructed by MEGA11.0 software with Neighbor-joining analysis using 1000 times bootstrap replications.

### 2.5. Preparation of Sample

#### 2.5.1. Bacterial Cell Resuspension

Overnight precultures of strains (10^9^ CFU/mL) were collected by centrifuging at 6000× *g* for 10 min, and the supernatant was removed. The precipitate obtained from centrifugation was subjected to washing three times using a sterile phosphate-buffered saline (PBS) solution (pH 6.8). Subsequently, the strain cell was suspended in PBS and its concentration was adjusted to 1.0 × 10^8^ CFU/mL.

#### 2.5.2. Cell-Free Supernatants and Extracts

The cell-free supernatant (CFS) was harvested by centrifugation at 8000× *g* for 15 min. The intact cells were washed three times with 0.1 mol/L sterile PBS solution (pH 6.8), and the cells were resuspended in PBS and adjusted to 1.0 × 10^9^ CFU/mL, which were incubated at 37 °C for 12 h and centrifuged again to remove cells. Then, the cell-free extracts (CFE) were acquired using ultrasonication, employing 3–5 s pulses for 15 min in an ice bath. The cellular components were harvested by centrifuging at 12,000× *g* for 10 min. The CFS and CFE were treated with filtration using 0.22 μm filter membranes to remove any intact bacterial cells. The filtered samples were then stored at −80 °C for subsequent experiments.

### 2.6. Determination of Hydrophobicity

The bacterial resuspension served as the control group, and the strain’s affinity for hydrocarbons was used to reflect the hydrophobicity of the strain. One milliliter of chloroform and ethylacetate were added to 4 mL of bacterial resuspension and vortexed for 2 min. The water phase was absorbed after layering at 37 °C for 30 min and the OD600 was measured. The hydrophobic ability of the strain was calculated according to the Equation, as follows.

$$Hydrophobic\left(\%\right)\equiv (1-\frac{{OD}_{sample}}{{OD}_{control}})\times 100$$

### 2.7. Auto-Aggregation Measurement

Four milliliters of the bacterial resuspension were vortexed for 2 min and the OD600 was tested. Then, the remaining bacterial resuspension was placed at 37 °C, and the supernatant was taken at 3 h, 12 h and 24 h to measure the OD600. The auto-aggregation was calculated as follows:

$$Auto-aggregation\left(\%\right)\equiv (1-\frac{{OD}_{supernatant}}{{OD}_{resuspension}})\times 100$$

### 2.8. Gastrointestinal Tolerance Test

The tolerance of strains to artificial gastrointestinal fluid was evaluated as described by Ranadheera et al. [24]. In 20 mL PBS (pH 7.2), 0.06 g pepsin (*w*/*v*, 0.3%) was added and the pH was adjusted to 3.0, and then the solution was filtered with 0.22 μm microporous membrane to obtain artificial gastric fluid. An amount of 0.06 g trypsin (*w*/*v*, 0.3%) and 0.2 g ox bile salt (*w*/*v*, 1%) were added to 20 mL PBS (pH 7.2), and the pH was adjusted to 8.0. Then, the solution was filtered through a 0.22 μm microporous membrane to obtain the artificial intestinal fluid. The strains were cultivated in 10 mL of MRS broth for 16 h at 37 °C. The quantity of viable cells was determined and recorded as N_0_. And 1 milliliter bacterial suspension was suspended in 5 mL artificial gastric fluid and incubated for 3 h at 37 °C. Then, the sample was centrifuged at 8000 r/min for 10 min, and, after the supernatant was removed, four milliliters of artificial intestinal fluid was added and incubated at 37 °C for 8 h. The viable cell count (N) was assessed, and the survival rate was calculated by comparing N with N_0_ using the following method:

$$Survival\;rate\left(\%\right)=\frac{logN}{logN_{0}}\times 100\%$$

### 2.9. Inhibitory Activity Assay of α-Glucosidase and DPP-IV

#### 2.9.1. α-Glucosidase Inhibitory Activity

The activity of α-glucosidase inhibitory activity was measured according to Cui et al. [25] with some modifications. The α-glucosidase inhibitory activity was measured by mixing 25 μL of the samples (CFS or CFE) with 50 μL of α-glucosidase (0.01 U/mL) in PBS (0.1 M, pH 6.8) and then incubating the mixture at 37 °C for 10 min. Subsequently, fifty microliters of a 4-Nitrophenyl-β-D-glucopyranoside (PNPG) solution with a concentration of 0.01 U/mL were introduced into each well. The resulting mixture was then subjected to incubation at a temperature of 37 °C for 15 min. At last, one hundred microliters of a 0.1 M Na_2_CO_3_ solution was added to stop the process. The OD405 before and after the reaction was determined. The α-glucosidase inhibitory activity was calculated using the following formula:

$$Inhibition\;rate\left(\%\right)=(1-\frac{A_{1}-A_{2}}{A_{3}-A_{4}})\times 100$$

In the formula:

A1: PNPG + sample + α-Glucosidase + Na_2_CO_3_.
A2: PNPG + sample + PBS (0.1 mol/L, pH 6.8) + Na_2_CO_3_.
A3: PNPG + PBS (0.1 mol/L, pH 6.8) + α-Glucosidase + Na_2_CO_3_.
A4: PNPG + PBS (0.1 mol/L, pH 6.8) + PBS(0.1 mol/L, pH 6.8) + Na_2_CO_3_.

#### 2.9.2. DPP-IV Inhibitory Activity

The impact of the strains on DPP-IV activity was assessed using a modified method described by He [26]. In summary, twenty-five microliters of the bacterial sample (CFS or CFE) and 25 μL of gly-pro-p-nitroanilide (0.2 mM) were preincubated at 37 °C for 10 min in a 96-well microplate. Then, fifty microliters of DPP-IV (0.01 U/mL) was introduced, and the sample was incubated at 37 °C for 60 min. The reactions were terminated by the addition of 100 μL sodium acetate buffer (1 M, pH 4.0), and the absorbance of the samples was measured at 405 nm by spectrumMax i3X Microplate reader (MD, Shanghai, China). The DPP-IV inhibition rate (DIR) was calculated as follows:

$$Inhibition\;rate\left(\%\right)=(1-\frac{A_{1}-A_{2}}{A_{3}-A_{4}})\times 100$$

In the formula:

A1 (sample): Gly-pro-p-nitroanilide + sample + DPP-IV + sodium acetate buffer.
A2 (sample blank): Gly-pro-p-nitroanilide + sample + Tris-HCl buffer (100 mM, pH 8.0) + sodium acetate buffer.
A3 (positive control): Gly-pro-p-nitroanilide + Tris-HCl buffer (100 mM, pH 8.0) + DPP-IV + sodium acetate buffer.
A4 (negative control): Gly-pro-p-nitroanilide + Tris-HCl buffer (100 mM, pH 8.0) + Tris-HCl buffer (100 mM, pH 8.0) + sodium acetate buffer.

### 2.10. Antioxidant Activities

#### 2.10.1. Reducing Activity

The activity of reduction was evaluated as previously described with some modifications [27]. A mixture was prepared by combining 1 mL of samples (CFS or CFE), 1 milliliter of potassium ferricyanide (10 g/L) solution and 1 milliliter of PBS (0.2 M, pH 6.6) solution. The mixture was incubated at 50 °C in the dark for 20 min, and then one milliliter of 100 g/L of trichloroacetic acid (TCA) was added to the mixture. The solution was subsequently centrifuged for 10 min at 4000× *g*, and then 1 mL of the supernatant was mixed thoroughly with 1 mL of distilled water and 0.4 mL of ferric chloride (1.0 g/L). The measurement of absorbance was conducted at a wavelength of 700 nm, and the reducing activity was converted to cysteine equivalent.

#### 2.10.2. DPPH Free Radical-Scavenging Activity

The DPPH free radical-scavenging capacity of strains was determined using a modified method as previously described [28]. A 1.5 mL sample (CFS or CFE) with 1.5 mL DPPH absolute ethanol solution (0.2 mM), absolute ethanol solution or distilled water was mixed. After being placed in the dark for 60 min, the supernatant was harvested by centrifuging at 6000× *g* for 10 min, and the absorbance was measured at 517 nm. The scavenging ability was calculated as follows:

$$Scavenging\;Activity\left(\%\right)=(1-\frac{A_{DPPH}-A_{Ethanol}}{A_{Distilledwater}})\times 100$$

#### 2.10.3. Hydroxyl Radical-Scavenging Activity

The capacity to scavenge hydroxyl radicals was assessed using a previously established assay [29]. The reaction mixture comprised 1.0 mL of 1,10-phenanthroline solution (0.75 mM), 2.0 mL of PBS (pH 7.4), 1.0 mL of samples (CFS or CFE) and 1.0 mL of FeSO_4_ (0.75 mM). Subsequently, the reaction was commenced by adding 1.0 mL of 6 mM H_2_O_2_ and incubated at 37 °C for 90 min. The solution’s absorbance was determined at a wavelength of 536 nm. A blank group was established by substituting the sample with distilled water, and a control group was established by substituting the hydrogen peroxide solution with distilled water. The hydroxyl radicals’ scavenging capacity was quantified as follows:

$$Scavenging\;Activity\left(\%\right)=\frac{A_{Sample}-A_{Blank}}{A_{Control}-A_{Blank}}\times 100$$

#### 2.10.4. Superoxide Anion Radical-Scavenging Activity

The superoxide anion radical-scavenging ability was evaluated following the method of Rwubuzizi [30]. We mixed 2.8 mL of 0.05 M Tris-HCl buffer solution (pH 8.2) with 0.1 mL of the sample (CFS or CFE) in a 10 mL cuvette, and then incubated for 20 min at room temperature (25 °C). After adding 0.1 mL of o-benzenetriol (0.05 M), the mixture was vortexed and placed in the dark at 25 °C for 5 min. The reaction was terminated by adding 1 mL of HCl solution (8 M), and the absorbance was measured at a wavelength of 320 nm. The control group was set by replacing the sample with plasma water, and scavenging activity was calculated with the following formula:

$$Scavenging\;Activity\left(\%\right)=(1-\frac{A_{Sample}}{A_{Control}})\times 100$$

#### 2.10.5. Lipid Peroxidation Inhibiting Capacity

The assessment of lipid peroxidation inhibition capacity was performed using the method previously reported by Hsu, with minor adjustments [31]. In summary, a mixture was prepared by combining 0.5 mL of PBS (pH 7.0), 1.0 mL of linoleic acid emulsion, 0.2 mL of FeSO_4_ (0.01%), 0.2 mL of ascorbic acid (0.01%) and 0.5 mL of samples (CFS or CFE). The mixture was then incubated at 37 °C for 12 h. After incubation, two milliliters of reaction solution with 0.2 mL TCA (0.4%), 2.0 mL TBA (0.8%) and 0.2 mL BHT (0.4%) were added, then vortexed for 1 min. The mixture was incubated at 100 °C for 30 min and cooled. Then, two milliliters of chloroform were added and centrifuged at 4000× *g* for 10 min. The absorbance of the supernatant was measured at 532 nm wavelength. The blank control group was set instead of the sample with PBS and the calculation formula is as follows:

$$Inhibiting\;effect\left(\%\right)=\frac{{A_{Blank}-A}_{Sample}}{A_{Blankl}}\times 100$$

#### 2.10.6. Principal Component Analysis (PCA)

Due to the different antioxidant mechanisms of different strains, the antioxidant level of the strains could not be determined by the results of a single antioxidant activity assay. Therefore, based on the results of each antioxidant activity assay, different antioxidant indexes were subjected to principal component analysis using SPSS 25.0. Then, ORIGIN 21.0 was applied to obtain the principal component loading plot and factor score plot, and the final score was calculated to observe the relationship between different antioxidant activities and the combined antioxidant activity of different strains and to screen strains with strong antioxidant activity.

### 2.11. GLP-1 Secretion Assay

The STC-1 cell line was inoculated in 6-well plates and cultured until the density reached 2 × 10^5^ per well and the cell culture reached 80% confluence. Then, the cells were washed twice with Ca^2+^- and Mg^2+^-free PBS buffer, and cultured in glucose- and L-glutamine-free DMEM for 30 min. The strain was washed twice with PBS and centrifuged at 8000 g/min for 10 min to collect the precipitate, the bacteria cells were suspended in the above DMEM medium, and the concentration of the bacterial cells solution was adjusted to 10^8^ CFU/mL, then incubated at 37 °C for 4 h. The supernatant was collected in a 1.5 mL centrifuge tube and centrifuged at 5900× *g* for 10 min to remove the cell precipitation, and the GLP-1 content was determined according to the method of the ELISA kit, and the supernatant of STC-1 cells without *lactobacilli* inoculation was used as a blank control.

### 2.12. qRT-PCR

According to the instructions of the RNA extraction kit, total cellular RNA was extracted, RNA concentration was determined, and the detected genes and related primers were shown in Table 2.

### 2.13. Statistical Analysis

Data are expressed as the mean ± standard deviation (*n* = 3 independent experiments). The statistical significance of the difference was determined using a one-way analysis of variance (ANOVA, SPSS 25.0). Values of *p* < 0.05 were considered to be statistically significant. GraphPad Prism 9.5, Origin 21.0 and Excel 2021 were used for charting.

## 3. Results

### 3.1. Characterization of Isolated Strains

**Figure 1:**
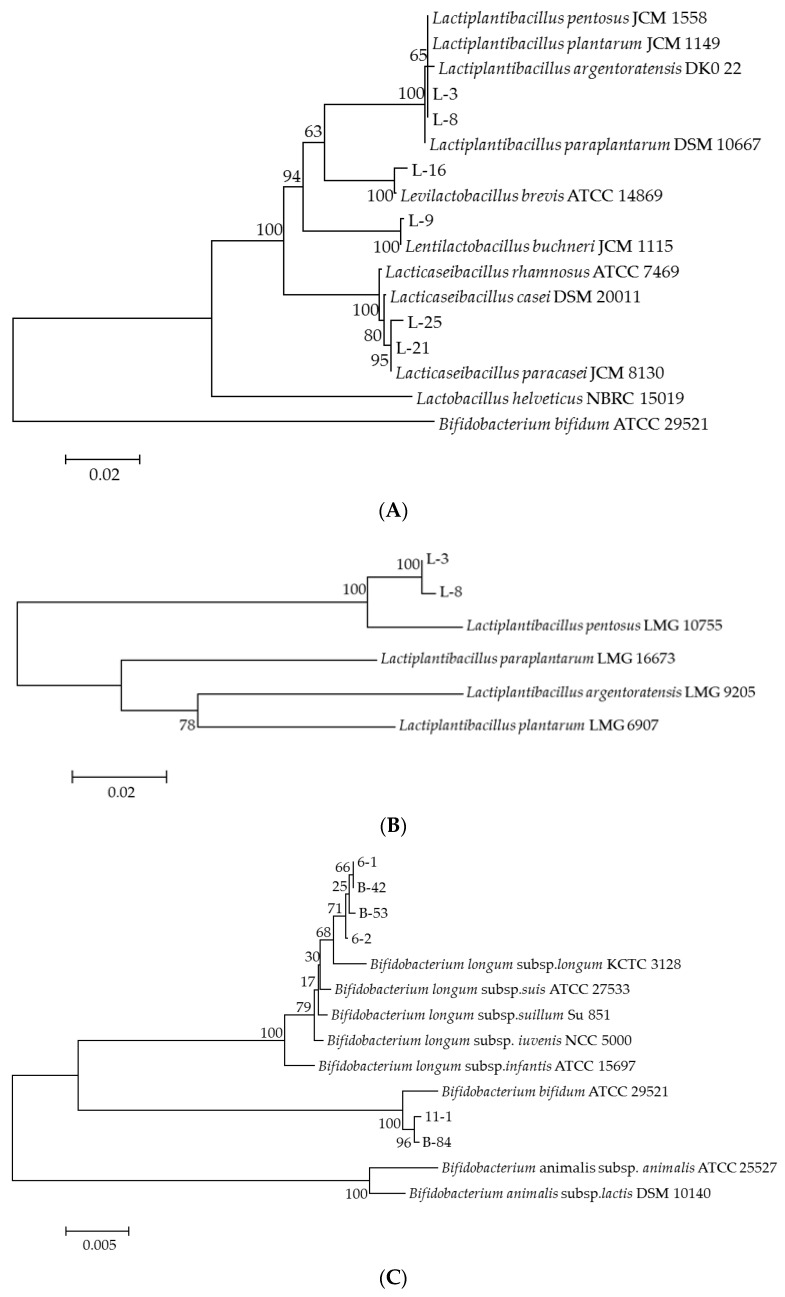

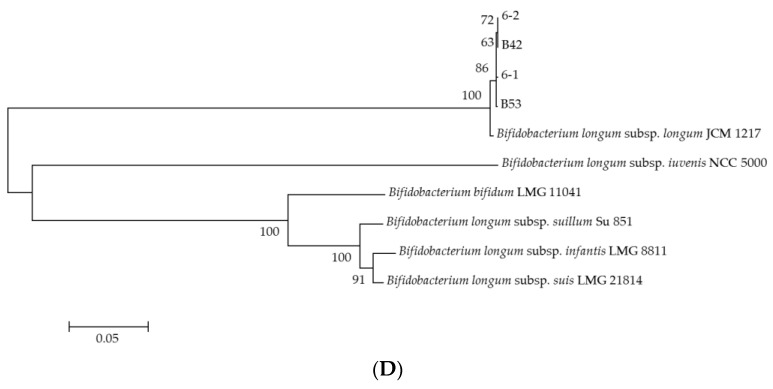
Neighbor-joining tree showing the phylogenetic relationships of isolated strains and related type strains based on different gene sequences and 1000 times bootstrap replications was used in the analysis. (**A**) Phylogenetic tree of *Lactobacilli* using 16S rRNA gene sequences, Bar, 0.02 substitutions per nucleotide position. (**B**) Phylogenetic tree of *Lactobacilli* using *pheS* gene sequences, Bar, 0.05 substitutions per nucleotide position. (**C**) Phylogenetic tree of *Bifidobacterium* 16S rRNA gene sequences, Bar, 0.005 substitutions per nucleotide position. (**D**) Phylogenetic tree of *Bifidobacterium* using *ClpC* gene sequences, Bar, 0.05 substitutions per nucleotide position.

Six strains of *Lactobacilli* and six strains of *Bifidobacterium* were isolated from healthy children 3 years old. The phylogenetic trees of isolated strains based on 16S rRNA, *pheS* and *ClpC* gene sequences are shown in Figure 1. From Figure 1A, it can be seen that strains L-21 and L-25 are located in the same phylogenetic branch as the type strain of *Lacticaseibacillus paracasei*, strains L-16 and L-9 are located in the same phylogenetic branch as the type strains of *Levilactobacillus brevis* and *Lentilactobacillus buchneri*, respectively, while strains L-3 and L-8 are located in the same phylogenetic branch as the type strains of *Lactiplantibacillus pentosus*, *Lactiplantibacillus plantarum* and *Lactiplantibacillus argentoratensis*. Current research indicates that the sequence homology of the 16S rRNA gene sequence of *Lactiplantibacillus pentosus* and *Lactiplantibacillus plantarum* is consistent; therefore, further experiments are needed to determine the taxonomic status of strains L-3 and L-8. From Figure 1B, it can be seen that strains L-3 and L-8 are located in the same phylogenetic branch as the type strains of *Lactiplantibacillus pentosus*. Therefore, combined with the result of the 16S rRNA gene sequence analysis, strains L-3 and L-8 are identified as *Lactiplantibacillus pentosus*. From Figure 1C, it can be seen that strains 11-1 and B-84 are located in the same phylogenetic branch as the type strains of *Bifidobacterium bifidum*, while strains 6-1, 6-2, B-42 and B-53 are all located in the same phylogenetic branch as the 5 subspecies of type strains of *Bifidobacterium longum*. Therefore, further experiments are needed to determine the taxonomic status of strains 6-1, 6-2, B-42 and B-53. From Figure 1D, it can be seen that strains 6-1, 6-2, B-42 and B-53 are located in the same phylogenetic branch as the type strains of *Bifidobacterium longum* subsp. *longum*. Therefore, combined with the result of the 16S rRNA gene sequence analysis, strains 6-1, 6-2, B-42 and B-53 are identified as *Bifidobacterium longum* subsp. *longum*.

### 3.2. Hydrophobicity of Isolated Strains

**Figure 2:**
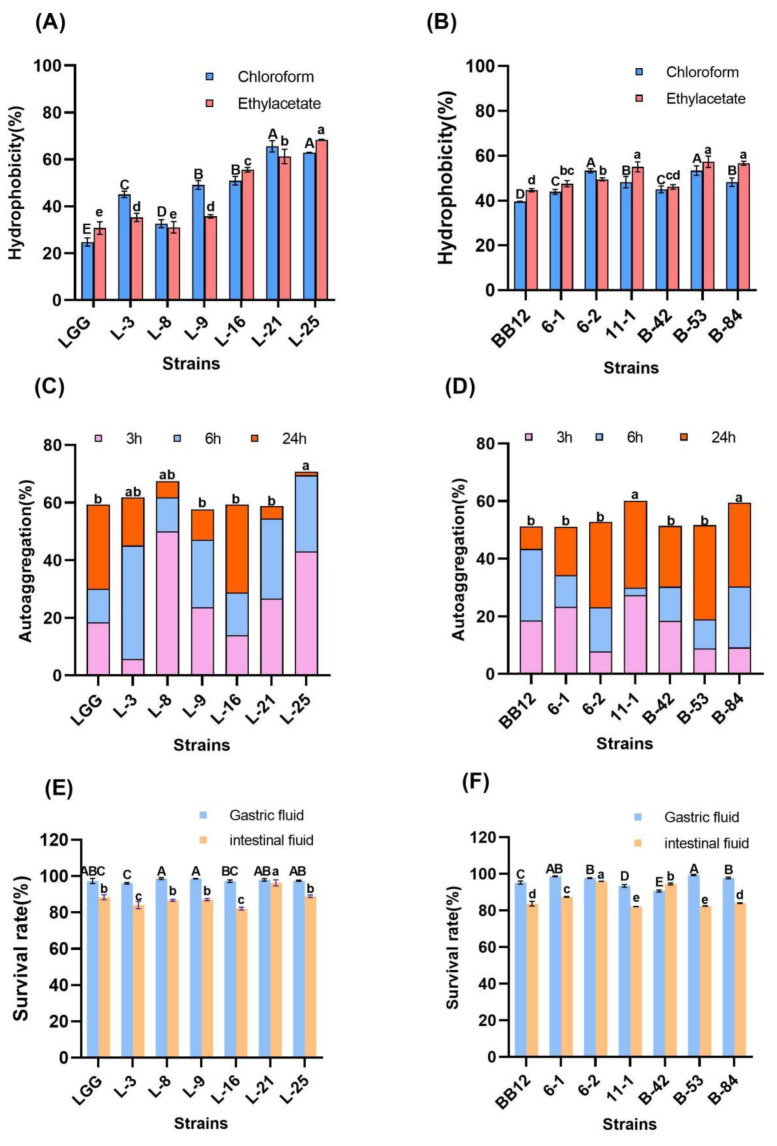
Comparison of *Lactobacilli* and *Bifidobacteria* for their hydrophobicity, auto-aggregation and gastrointestinal fluid tolerance. (**A**,**B**) Hydrophobicity of *Lactobacilli* and *Bifidobacteria*. (**C**,**D**) Auto-aggregation of *Lactobacillus* and *Bifidobacteria*. (**E**,**F**) Gastrointestinal fluid tolerance of *Lactobacilli* and *Bifidobacteria*. Among these, (**A**,**C**,**E**) characterize *Lactobacilli* and (**B**,**D**,**F**) characterize *Bifidobacteria*. Significant differences (*p* < 0.05) among different groups are indicated with different superscript lowercase and uppercase letters.

It is currently considered that strains with hydrophobicity greater than 30% might perform good adhesion and colonization [32]. The hydrophobicity levels of the fourteen selected strains are shown in Figure 2A,B. In chloroform, the hydrophobicity of isolated *Lactobacilli* strains was from 32.61% to 65.55% (Figure 2A). It was interesting to note that all strains had higher hydrophobicity than that of the reference strain LGG, which was 24.77% (*p* < 0.05). Among them, the hydrophobicity of L-21 and L-25 is significantly higher than that of other strains, which was 65.55% and 62.86%, respectively (*p* < 0.05). A similar situation was found in ethylacetate. L-25 and L-21 had higher hydrophobicity than the other strains, which were 68.31% and 61.22%, respectively (*p* < 0.05). As shown in Figure 2B, the hydrophobicity of each *Bifidobacterium* strain was higher in ethylacetate than in chloroform. The hydrophobicity of 6-2 (53.32%) and B-53 (53.37%) in chloroform were significantly above that of the remaining strains (*p* < 0.05). In ethylacetate, 11-1 (55.05%), B-53 (57.28%) and B-84 (56.64%) had significantly higher hydrophobic than the others (*p* < 0.05). Notably, reference strain BB12 (39.52%) also has the lowest hydrophobicity among all the *Bifidobacterium* strains. In summary, all strains have excellent hydrophobicity (>30%), which helps the bacteria to adhere and colonize in the host intestinal tract.

### 3.3. Auto-Aggregation of Isolated Strains

The probiotic strains can adhere to intestinal epithelial cells and mucosal surfaces, thereby reducing the colonization of pathogens by competing for ecological niches. Normally, the aggregation ability of bacterial cells is positively associated with cell adherence ability [33]. In this research, the reference strains and the 12 identified strains were tested for their ability to auto-aggregate at 3 h, 6 h and 24 h, respectively (Figure 2C,D). The results showed that the auto-aggregation rate of all strains exceeded 50% after incubation for 24 h. The auto-aggregation percentage of L-3 was highly variable at 3–6 h, which increased by 39.41%. After 6 h of incubation, L-25 exhibited a higher auto-aggregation percentage of 69.47% and reached its peak (70.76%) after 24 h of incubation, which even surpassed that of the reference strain LGG (*p* < 0.05). It is worth noting that L-25 showed a rapid self-polymerization, and its auto-aggregation rate nearly did not change during the incubation period from 6 h to 24 h, which might benefit its rapid colonization in the host intestinal tract. The auto-aggregation results of *Bifidobacteria* were illustrated in Figure 2D, 11-1 and BB12 showed higher auto-aggregation rates of 27.35% after incubation for 3 h and 43.41% after incubation for 6 h, respectively. Especially after 24 h, the auto-aggregation rate of 11-1 was the highest, reaching 60.05%. In contrast, 6-1 showed the lowest auto-aggregation rate of 51.07%. Except for BB12, the auto-aggregation rate of other *Bifidobacteria* strains changed greatly during the incubation period from 6 h to 24 h, especially the auto-aggregation rate of B-53, which increased by 32.76% during this period.

### 3.4. Gastrointestinal Fluid Tolerance

Gastrointestinal fluid tolerance of the *Lactobacilli* and *Bifidobacteria* strains was assessed in vitro (Figure 2E,F). All the *Lactobacilli* tested showed a high tolerance with a survival rate range from 96.1% to 98.65% in artificial gastric fluid, and there was no significant difference compared with the reference strain LGG (*p* > 0.05). In artificial intestinal fluid, the survival rate of L-21 was 96.27%, which was significantly higher than other strains (*p* < 0.05). The gastrointestinal fluid tolerance results of *Bifidobacteria* showed that B-53 had the best tolerance to gastric fluids with a survival rate of 99.41%, while 6-2 had the best tolerant ability to artificial intestinal fluids with a survival rate of 94.49%. Furthermore, all strains showed excellent gastrointestinal fluid tolerance, especially L-21 and 6-2, with a survival rate above 94%, which could favor their probiotic effects in vivo.

### 3.5. Hypoglycemic Potential of Isolated Strains

#### 3.5.1. α-Glucosidase Inhibitory Activity

**Figure 3:**
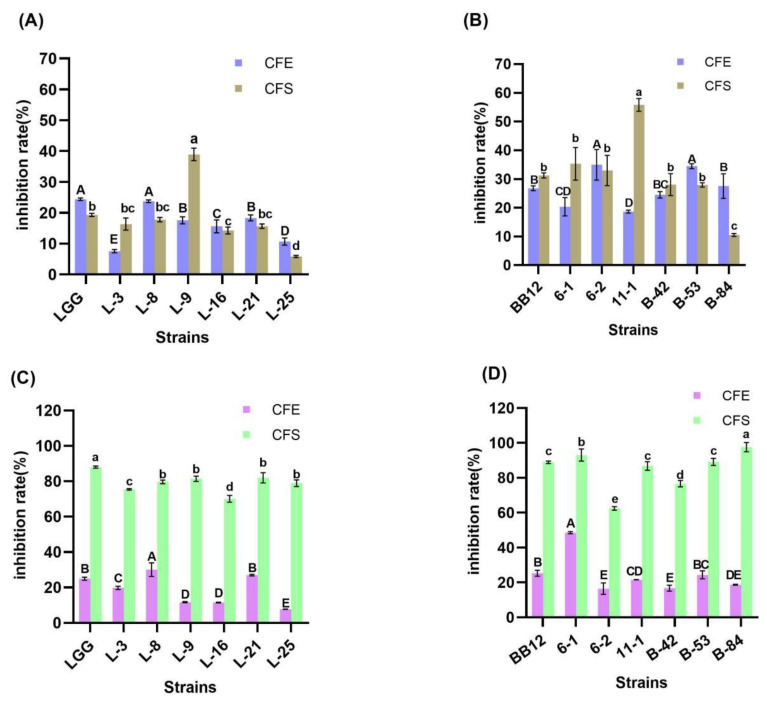
α-Glucosidase inhibitory activity and DPP-IV inhibitory activity of strains. (**A**) α-Glucosidase inhibitory activity of *Lactobacilli*. (**B**) α-Glucosidase inhibitory activity of *Bifidobacteria*. (**C**) DPP-IV inhibition activity of *Lactobacillus*. (**D**) DPP-IV inhibition activity of *Bifidobacteria*. Significant differences (*p* < 0.05) among different groups are indicated with different superscript lowercase and uppercase letters.

Alpha-glucosidase is a target for drug development that inhibits carbohydrate absorption and lowers blood sugar [34]. Therefore, inhibition of intestinal α-glucosidase activity suppresses hydrolysis of polysaccharides and disaccharides, lowering blood glucose levels [35]. The inhibitory activity of α-glucosidase of the *Lactobacillus* and *Bifidobacteria* strains is shown in Figure 3. The inhibitory activity of the CFE of *Lactobacillus* ranged from 7.53% to 24.37%, and that of the CFS of *Lactobacillus* was between 5.85% and 38.91%. Among the CFE of *Lactobacillus*, the inhibitory activity of α-glucosidase of LGG was the highest with a value of 24.37%, followed by L-8 with a value of 23.76%. And those of the CFE of L-3 and L-25 were significantly lower than that of other strains (*p* < 0.05). In particular, the α-glucosidase inhibitory activity of CFS of strain L-25 was the lowest, and that of CFE of L-25 was also low, indicating limited α-glucosidase inhibitory activity. In Figure 3B, the α-glucosidase inhibitory activities of CFE and CFS of *Bifidobacteria* ranged from 18.63% to 34.95% and 10.53% to 55.78%, respectively, among which the α-glucosidase inhibitory activities of CFE of 6-2 and B-53 were significantly higher than other strains (*p* < 0.05). It is regrettable that although the α-glucosidase inhibitory activity of CFS of strain 11-1 was the significantly highest (*p* < 0.05), the α-glucosidase inhibitory activity of its CFE was the lowest among all the strains. Compared to the reference strain LGG and BB12, L-8,6-2 and B-53 exhibited excellent inhibitory activities of their CFS and CFE. Therefore, these strains showed potent α-glucosidase inhibitory activity.

#### 3.5.2. DPP-IV Inhibitory Activity

The DPP-IV inhibitory activities of the strains are shown in Figure 3C,D. The CFE of the *Lactobacillus* ranged from 7.71% to 30.08%, with LGG and L-8 showing significantly higher levels of inhibition (*p* < 0.05), while L-25 exhibited the lowest inhibition activity. The results of DPP-IV inhibitory activity of CFS showed that LGG was significantly the highest (*p* < 0.05). In contrast, both CFS and CFE DPP-IV inhibitory activity of L-16 were unsatisfactory and not suitable for further study. The CFE of *Bifidobacteria* ranged from 16.42% to 48.52%, and the most excellent inhibitory activity (*p* < 0.05) was found in 6-1, which was 48.52%. The CFS of *Bifidobacteria* performed higher DPP-IV inhibitory potential, such as B-84, and, except for 6-2, all other strains exceeded 70%. Meanwhile, the DPP-IV inhibitory activity of CFE of B-42 was insufficient and not significantly different from that of CFE of 6-2 (*p* > 0.05). In short, the DPP-IV inhibitory activities of CFS of LGG, L-8 and B-84 were significantly superior to the rest of the test strains in the same group. Moreover, 6-2 and B-42 were not chosen to be further investigated.

### 3.6. Antioxidative Activity of Isolated Strains

#### 3.6.1. Reducing Activity

**Figure 4:**
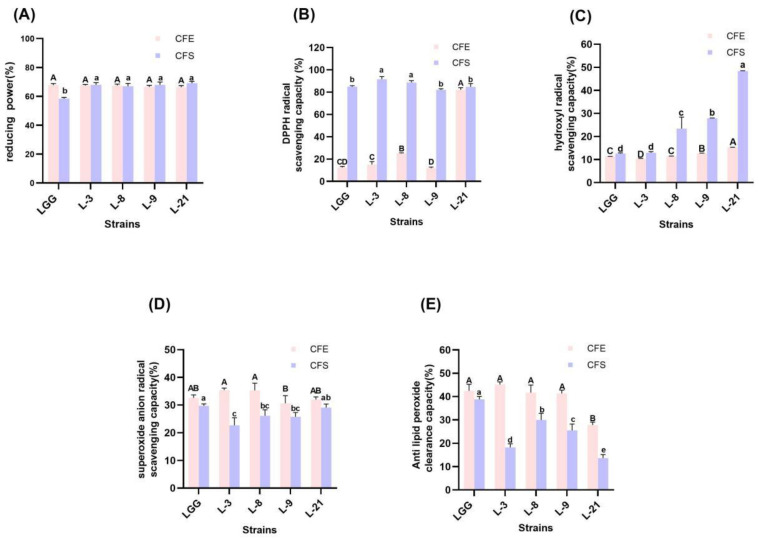
Antioxidative activity of *Lactobacillus*. (**A**) Reducing activity. (**B**) DPPH free radical-scavenging ability. (**C**) Hydroxyl radical-scavenging ability. (**D**) Superoxide anion radical-scavenging ability. (**E**) Lipid peroxidation inhibition capacity. Significant differences (*p* < 0.05) among different groups are indicated with different superscript lowercase and uppercase letters.

**Figure 5:**
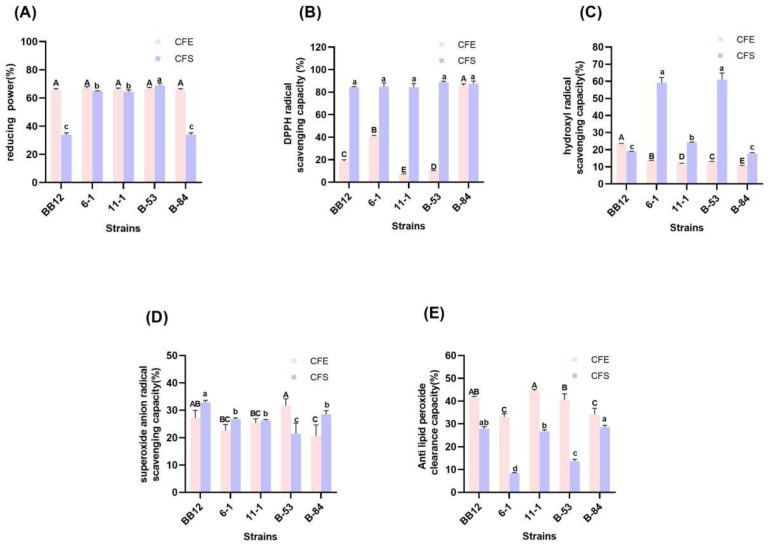
Antioxidative activity of *Bifidobacterium*. (**A**) Reducing activity. (**B**) DPPH radical-scavenging activity. (**C**) Hydroxyl radical-scavenging activity. (**D**) Superoxide anion radical-scavenging activity. (**E**) Lipid peroxidation inhibition activity. Significant differences (*p* < 0.05) among different groups are indicated with different superscript lowercase and uppercase letters.

The antioxidant activity of *Lactobacilli* and *Bifidobacteria* are shown in Figure 4 and Figure 5, respectively. Strains exhibited varying degrees of reduced activity. The reducing power of CFE of 5 strains of *lactobacilli* all exceeded 60% and there was no significant difference (*p* > 0.05). The CFS of LGG exhibited a reducing activity of 58.39%, which was significantly lower than that of other strains (over 60%), and the rest of the strains showed no significant difference in reducing activity (*p* > 0.05). The reducing activity of *Bifidobacterium* was shown in Figure 5A, there was no significant difference in the reducing activity of CFEs of each *Bifidobacterium* (*p* > 0.05), and the CFS of B-53 possessed the significantly highest reducing activity of 69.04% (*p* < 0.05).

#### 3.6.2. DPPH Radical-Scavenging Activity

In this study, all tested strains exhibited distinct DPPH radical-scavenging activity (Figure 4B and Figure 5B). The CFE of L-21 had the highest DPPH radical-scavenging activity of 82.09%, while that of L-9 exhibited the lowest DPPH radical-scavenging activity of 11.87%. Moreover, the CFS of all the strains exhibited higher DPPH radical-scavenging activity than that of their CFE, and among them, L-3 and L-8 have significantly higher DPPH radical-scavenging ability than the others (*p* < 0.05). In Figure 5B, the DPPH radical-scavenging ability of CFE of B-84 was significantly higher than that of other strains (*p* < 0.05). And among them, strain 11-1 had the lowest DPPH radical-scavenging activity of 7.37%. Furthermore, the DPPH radical-scavenging activity of CFS of each *Bifidobacterium* exceeded 80%, and there was no significant difference (*p* > 0.05).

#### 3.6.3. Hydroxyl Radical-Scavenging Ability

The CFE and CFS of all strains could eliminate the hydroxyl radical (Figure 4C and Figure 5C). The hydroxyl radical-scavenging rates of the CFE and CFS of the five *Lactobacilli* strains ranged from 10.37% to 15.32% and 12.61% to 48.39%, respectively. Among all the strains, the CFE of L-21 had the highest hydroxyl scavenging rate of 48.39% (*p* < 0.05), while the CFS of LGG had the lowest hydroxyl scavenging rate of 12.61% (*p* < 0.05). The hydroxyl radical-scavenging ability of *Bifidobacteria* was shown in Figure 5C. The CFE and CFS hydroxyl radical-scavenging rates of the five *Bifidobacterium* strains ranged from 10.58% to 23.56% and 17.81% to 60.94%, respectively. The CFE of BB-12 had the highest hydroxyl radical-scavenging rate of 23.56%; however, its CFS has a lower hydroxyl radical-scavenging rate of 19.04%, while the CFS of 6-1 and B-53 showed a significantly higher hydroxyl radical-scavenging capacity of 59.13% and 60.94%, respectively (*p* < 0.05).

#### 3.6.4. Superoxide Anion Radical-Scavenging Ability

In this study, there was no significant difference in the superoxide anion radical-scavenging rate of CFE between LGG and the other *Lactobacilli* strains (*p* > 0.05), and among all the strains L-8 showed the highest superoxide anion radical-scavenging rate of 35.40% (Figure 4D). LGG has the highest CFS superoxide anion radical-scavenging rate of 29.69%, and that of L-9 was 25.75% which was the lowest. However, among *Bifidobacteria*, only CFE of B-53 has the highest superoxide anion scavenging rate of 31.63%, yet its CFS had the lowest superoxide anion scavenging rate of 21.39%. Instead, the superoxide anion scavenging rate of CFS of BB-12 was 32.84%, which was significantly higher than other *Bifidobacteria* strains (*p* < 0.05).

#### 3.6.5. Lipid Peroxidation Inhibition Capacity

The lipid peroxidation inhibition capacity of strains is shown in Figure 4E. Apart from L-21, there was no significant difference in the lipid peroxidation inhibition capacity of CFE between LGG and the other *Lactobacilli* strains (*p* > 0.05), while the CFS of LGG showed significantly highest lipid peroxidation inhibition ability (*p* < 0.05). About the *Bifidobacterium* strains (Figure 5E), the lipid peroxidation inhibition capacity of the CFE of each strain was higher than that of the CFS of the same strain. And there was no significant difference in the lipid peroxidation inhibition capacity of CFE and CFS between BB-12 and 11-1 (*p* > 0.05).

#### 3.6.6. Principal Component Analysis

**Figure 6:**
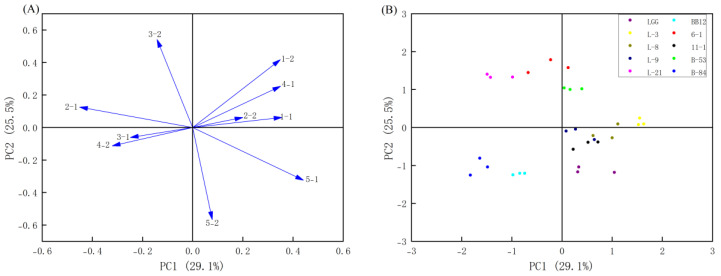
Loading and score plots of principal component analysis. (**A**) Loading plot for results of five different antioxidant assays. The main antioxidant components are displayed as “Antioxidant Index and Sample”. Antioxidant index numbers 1 to 5 represent reducing activity, DPPH free radical-scavenging ability, hydroxyl radical-scavenging ability, superoxide anion radical-scavenging ability, and lipid peroxidation inhibition capacity, respectively. Sample numbers 1 and 2 represent CFE and CFS, respectively. (**B**) Loading plot for results of five different antioxidant assays.

In this study, PCA was employed to assess the antioxidative activity of all strains. It was demonstrated in Figure 6A that PC1 accounting for 29.1% of the overall variance was distinguished by the decline in DPPH free radical-scavenging ability of CFE, the reduction in superoxide anion radical-scavenging ability of CFS, and the suppression of lipid peroxidation capacity of CFE. PC2 represents 25.5% of the overall variance, primarily correlated with the lipid peroxidation inhibition capacity of CFS, hydroxyl radical-scavenging ability of CFS and reducing the activity of CFS. Figure 6B displays the plot of strain scores for PC1 versus PC2. Furthermore, the overall score was utilized to categorize all examined strains (Table 3). The cumulative scores of L-9, L-21, B-53 and 6-1 were higher than those of other strains, with values of 0.29, 0.23, 0.54 and 0.31, respectively. It is essential to mention that the overall antioxidant activity of all strains of *Lactobacilli* and *Bifidobacterium* was more significant than the control strains in each group. Thus, these strains were chosen for further investigation based on the obtained results.

### 3.7. Stimulation GLP-1 Secretion of STC-1 Cells by Lactobacilli and Bifidobacterium Strains

**Figure 7:**
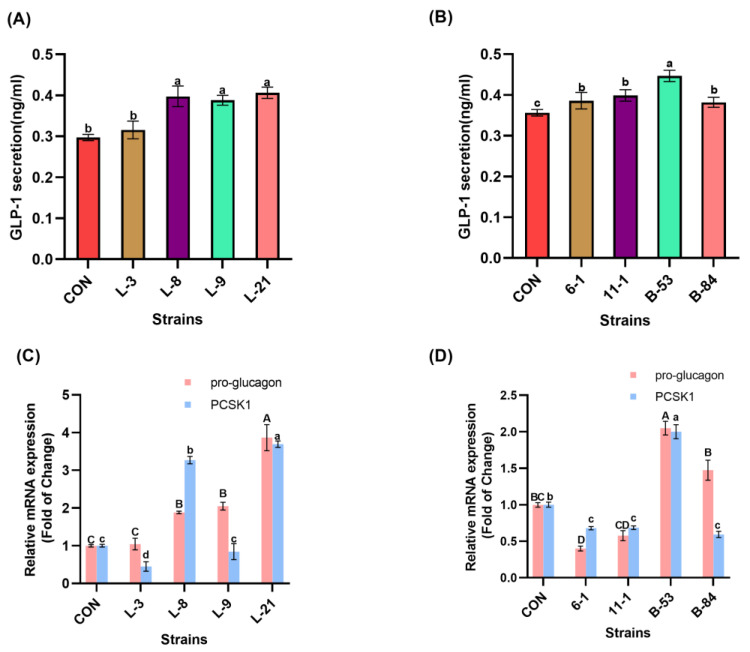
GLP-1 secretion and mRNA expression of related genes after *Lactobacilli* and *Bifidobacterium* action on STC-1 cells. (**A**) *Lactobacilli* stimulates STC-1 to secrete GLP-1 amount. (**B**) *Bifidobacterium* stimulates STC-1 to secrete GLP-1 amount. (**C**) Pro-glucagon and PCSK1 gene-related mRNA expression of *Lactobacilli*. (**D**) Pro-glucagon and PCSK1 gene-related mRNA expression of *Bifidobacterium*. Significant differences (*p* < 0.05) among different groups are indicated with different superscript lowercase and uppercase letters.

The stimulation result of STC-1 to secrete GLP-1 by *Lactobacillus* and *Bifidobacterium* is shown in Figure 7. The GLP-1 secretion level of STC-1 cells treated with each selected *Lactobacilli* and *Bifidobacterium* strain was higher than that of LGG and BB-12. As shown in Figure 7A, STC-1 cells treated by L-21 had the highest secretion level of GLP-1 (0.41 ng/mL), followed by L-8 (0.40 ng/mL), L-9 (0.39 ng/mL) and L3 (0.32 ng/mL) (*p* < 0.05). While incubated with *Bifidobacteria* (Figure 7B), the GLP-1 secretion level of STC-1 cells treated by B-53 showed the highest value of 0.45 ng/mL, which was significantly higher than the other strains (*p* < 0.05). Among selected *Bifidobacterium* strains, STC-1 cells incubated with B-84 showed the lowest GLP-1 secretion level of 0.37 ng/mL. The PCSK1 gene is a rate-limiting gene that controls the secretion of GLP-1, and which can cleave the expression product of the pro-glucagon gene to obtain the corresponding fragment of GLP-1. In this study, the mRNA expression level of pro-glucagon and PCSK1 genes of strains were detected. Compared to the blank control (Figure 7C), L-21 had significantly higher mRNA expression related to pro-glucagon and PCSK1 genes than other *Lactobacilli* strains (*p* < 0.05). Moreover, among all the *Lactobacilli* strains, the GLP-1 secretion level of STC-1 was positively correlated with the mRNA expression of its pro-glucagon gene and PCSK1 gene. Nevertheless, it is noteworthy that the mRNA expression of the PCSK1 gene was significantly lower in both group 6-1, group 11-1 and group B84 than that of the control group (Figure 7D), while the GLP-1 secretion level of STC-1 incubated with these strains was significantly higher than that of the control group (*p* < 0.05). And there was no significant difference in the mRNA expression of the pro-glucagon gene between group 11-1, group B84 and the control group. These results indicated that the GLP-1 secretion level of STC-1 cells was not only related to the mRNA expression levels of pro-glucagon and PCSK1 genes but also to additional factors. In addition, the GLP-1 secretion level of STC-1 cells incubated with the B-53 was the highest, and the mRNA expression level of pro-glucagon and PCSK1 genes was also significantly higher than that of other *Lactobacilli* strains (*p* < 0.05). It was concluded from the above that L-21 and B-53 were more effective in promoting GLP-1 secretion from STC-1 cells.

## 4. Discussion

A growing body of evidence suggests that breast milk contains *Bifidobacteria* and human milk oligosaccharides, which can be transferred to infants through breastfeeding. *Bifidobacteria* produce metabolites by metabolizing human milk oligosaccharides, which contribute to developing infant intestinal microbiota diversity [36]. The gut microbiota in the feces of 3-year-old healthy children tends to be already stabilized and the abundance of probiotics is relatively high, making it a good source of functional probiotic strains. Thus, 6 strains of *Lactobacilli* and 6 strains of *Bifidobacterium* were isolated from 5 children around 3 years old in this study. It is widely known that *Lactobacilli* and *Bifidobacterium* have numerous subspecies, but it is difficult to accurately identify by 16S rRNA gene sequencing alone, while the housekeeping gene *pheS* of *Lactobacilli* and the housekeeping gene *ClpC* of *Bifidobacterium* were often used to distinguish them with greater accuracy [22,23]. Therefore, in this study, *Lactobacilli* and *Bifidobacterium* were characterized using 16S rRNA gene sequencing and two housekeeping gene sequencing, separately.

At present, *Lactobacillus rhamnosus* GG and *Bifidobacterium animalis* subsp. *lactis* BB12 are nearly the most widely commercialized strains in the food and pharmaceutical industry because of their probiotic functions based on lots of published research papers. Thus, many research works have used *Lactobacillus rhamnosus* GG as the reference strain of *Lactobacilli*, and *Bifidobacterium animalis* subsp. *lactis* BB12 strains as the control strain of *Bifidobacterium* [37,38,39]. Li et al., used BB12 as a reference strain to study how *Bifidobacterium animalis* subsp. *lactis* A12 prevents obesity-related dyslipidemia by affecting energy metabolism [40]. Zhong et al., screened new antidiabetic strains from 12 strains of *Lactobacillus plantarum* in vitro by using *Lactobacillus rhamnosus* GG (LGG) as a reference strain [41]. There are also many similar studies, and thus we also use LGG and BB12 as reference strains in this study.

The ability of probiotics to adhere and colonize in the intestinal tract was usually assessed by auto-aggregation and cell surface hydrophobicity assay. The greater the auto-aggregating power of probiotics, the more conducive to their adhesion and colonization in the host intestinal tract, which could in turn inhibit the adhesion of pathogenic bacteria and provide a protective effect on the host intestinal tract [42,43]. In this study, all strains were highly self-aggregating; therefore, all strains could adhere to the host intestinal tract within a short period. Cell surface hydrophobicity is associated with non-specific adhesion [44]. And a highly hydrophobic cell surface is an essential factor in preventing environmental interference during probiotic adhesion and colonization [45]. Previous research had proposed that the criterion for high cell surface hydrophobicity was that the hydrophobicity of the cell surface exceeded 30% [32]. In this study, all strains except for LGG showed a high cell surface hydrophobicity. Therefore, all the strains would have a good adhesion in vivo. The colonization of exogenous *Lactobacilli* in the host is influenced not only by their own traits, but also by environmental factors, such as the host’s dietary habits and the relationship of the *Lactobacilli* to the host’s original intestinal microbiota [46]. The host ingestion of different growth factors may help to change the structure of the original intestinal microbiota and was important for exogenous *Lactobacilli* and *Bifidobacterium* colonization in the body [47]. In addition, *Lactobacilli* that colonize the host for a short period of time had to co-survive with the host’s original intestinal microbiota in order to colonize better [48]. However, this study only identified the factors that favor colonization of the strain in vitro; thus, future in vivo validation of colonization will be required.

High tolerance to gastrointestinal fluids is necessary for the colonization of the probiotics in vivo [49]. Our studies have demonstrated that in artificial gastric juice with pH 3.0, the Colony Forming Unit (CFU) only decreased by less than 1 logarithmic unit for all strains, which is consistent with the results of Zou et al. [50], and much higher than the results in the study of Marco et al. [38]. Das et al. [51] showed that the survival rate of *Limosilactobacillus fermentum* TIU19 was more than 99% after being treated with trypsin (pH 8), suggesting that the challenge of artificial intestinal fluids on probiotics is essentially from bile salts. In this study, we found a decrease in CFU of less than 2 log units for all strains in the artificial intestinal fluid with 3% (*w*/*v*) bile salt content, and concerning the results of Hove et al. [52], all strains could be considered to have good tolerance to gastrointestinal fluids.

Previous studies have shown that the exopolysaccharide of LAB has α-glucosidase inhibitory activity [53]. In this study, the α-glucosidase inhibitory activity of CFS of all strains except L-16, L-25 and B-84 was higher than 15%. Interestingly, the α-glucosidase inhibitory activity of *Bifidobacterium* CFS was generally higher compared to *Lactobacilli* CFS and it might be assumed that the *Bifidobacterium* used in this test generally produced more extracellular polysaccharides than *Lactobacilli*. However, the α-glucosidase inhibitory activity of CFE was low for all strains. Pyclik et al., concluded that *Bifidobacterium* was a completely anaerobic bacterium that tended to secrete large amounts of polysaccharides into the extracellular space in order to encapsulate its cells and contribute to the maintenance of an anaerobic environment for the cytosol [54]. However, the stress caused by the accumulation of polysaccharides was not conducive to the sustained production of extracellular polysaccharides [55].

*Lactobacilli* and *Bifidobacterium* have been reported to possess DPP-IV inhibitory activity [56,57]. Studies have shown that some natural protein hydrolysis products have high DPP-IV inhibitory activity [57,58,59], and peptides and amino acids dominate natural DPP-IV inhibitors. According to the mechanism of DPP-IV degradation of GLP-1, peptides could act as substrates for DPP-IV, which could bind to DPP-IV and reduce the binding of GLP-1 to DPP-IV, thus prolonging the half-life of GLP-1. Our experimental results showed that the DPP-IV inhibitory activity of all strains of CFS was higher than 60% and much higher than that of CFE. Thus, we concluded that all strains might secrete a large amount of protein hydrolysis products during the growth process. However, the lower DPP-IV inhibitory activity of CFE might be due to the destruction of protein hydrolysis products caused during the ultrasonic wall-breaking process.

The metabolism of the human body in different internal and external environments will produce many reactive oxygen species, usually in the form of free radicals, which can cause serious harm to the human body [60]. Oxidative stress is also thought to be a significant characteristic in the development of diabetes. Consequently, the antioxidant activity of probiotics is also an essential part of its probiotic properties. Studies have shown that probiotics possess their own antioxidant enzyme system that produces metabolites with antioxidant activity, such as GSH, exopolysaccharides, butyric acid, and folic acid [61]. And the lipid peroxidation inhibition capacity of the same strain was much higher for CFE than for CFS, which was similar to the findings of Cai et al. [62]. It has been shown that higher reactive oxygen species in vivo affect probiotic colonization [58]. Therefore, it is necessary to reduce oxidative stress by screening strains with higher antioxidant activity. In our experiment, the antioxidant capacity of *Lactobacillus* and *Bifidobacterium* was further evaluated using principal component analysis to screen the strains with higher comprehensive antioxidant capacity. The results for DPPH scavenging capacity, hydroxyl radical-scavenging capacity and superoxide anion scavenging capacity were similar to those of Zhao et al. [63].

It is well known that GLP-1 helps alleviate T2DM [64]. In this experiment, STC-1 cells were incubated with probiotics to test their ability to secrete GLP-1, and thus strains with mitigating effects on T2DM were screened. Pro-glucagon and PCSK1 are critical genes that determine GLP-1 secretion [65,66], the cleavage of the expression product of the pro-glucagon gene is conducted by PCSK1 protein, and then the corresponding fragment for GLP-1 is generated [67]. Thereby, the PCSK1 gene is a rate-limiting gene that affects GLP-1 secretion. Our results also confirmed this phenomenon, as we found that after incubation of STC-1 cells with selected strains, there is a positive correlation between the GLP-1 secretion level and the mRNA expression level of pro-glucagon and PCSK1 genes in most strains. However, STC-1 cells incubated with B-84 had low expression levels of PSCK1, and it did not affect the secretion amount of GLP-1, which might be due to the effect of the PCSK2 gene [68]. The PCSK2 gene is mainly expressed in the pancreas, but it is also expressed to a certain extent in intestinal cells, and PCSK2 expressed in the intestine helps to improve the secretion of GLP-1 [69].

In summary, the candidate probiotic strains with anti-type II diabetes-related activities are currently obtained through in vitro experiments and have a good stimulating effect on GLP-1 secretion in STC-1 cells in this study. However, there are still great challenges in extrapolating the results of in vitro experiments to intact organisms. For example, in this study, we have performed in vitro research of auto-aggregation and cell surface hydrophobicity assay to test the adhesion capacity of isolated strains; however, the colonization of strains in the host is influenced not only by their own traits, but also by environmental factors, such as the host’s dietary habits and the genetic relationship of the exogenous strains to the host’s original intestinal microbiota. Thus, the ability of strains to adhere and colonize in the host intestinal tract still needs to be further confirmed in vivo. Moreover, the ability to stimulate GLP-1 secretion of isolated strains is only investigated in STC-1 cells, the internal environment of the human body is much more complex, and we cannot directly equate the results of cell experiments with those in vivo. Thus, further in vivo experiments to study the effects of *Lacticaseibacillus paracasei* L-21 and *Bifidobacterium longum* subsp. *longum* B-53 on ameliorating type II diabetes are needed, and the detailed mechanism of their improvement effect on anti-type II diabetes in vivo also needs to be confirmed before they could be used as antidiabetic strains served as a food supplement and drug ingredient.

## 5. Conclusions

In this study, six strains of *Lactobacilli* and six strains of *Bifidobacterium* were isolated and purified from the feces of healthy 3-year-old children. They all had better hydrophobicity, self-aggregation and gastrointestinal fluid tolerance than the control strains of LGG and BB-12, respectively. *Lactiplantibacillus plantarum* L-8 and L-3, *Lentilactobacillus buchneri* L-9, *Lacticaseibacillus paracasei* L-21, *Bifidobacterium bifidum* 11-1 and B-84, *Bifidobacterium longum* subsp. *longum* 6-1 and B53 had good α-glucosidase inhibitory activity and DPP-IV inhibitory activity, and the combined antioxidant activity of these eight strains was higher than that of LGG and BB-12, respectively. Moreover, *Lacticaseibacillus paracasei* L-21 and *Bifidobacterium longum* subsp. *longum* B-53 were the most effective strains among isolated *Lactobacillus* and *Bifidobacterium* strains, respectively, in promoting the intestinal L-cells to secrete GLP-1. Thus, they might be applied as potential antidiabetic strains that serve as food supplements and drug ingredients.

## Figures and Tables

**Table 1:** Primer sequences of PCR amplification of different genes.

Strain Name	Gene Name	Primer Sequence (5′ → 3′)
Primer for 16SrRNA gene amplification		
Lactobacilli	27F	AGAGTTGATCCTGGCTCAG
	1495R	CTACGGCTACCTTGTTAGA
Bifidobacteria	Bif285	GAGGGTTCGATCTGGCTCAG
	261	AAGGAGGTGATCCAGCCGCA
Primers for housekeeping gene amplification		
Lactobacilli	*pheS*(21 F)	CAYCCNGCHCGYGAYATGC
	*pheS*(23 R)	GCRTGRACCAVCCNGCHCC′
Bifidobacteria	*ClpC*(uni)	GATACCCAAGTACATCGAG
	*ClpC*(Rev)	CATCCTCATCGTCGAACAGAAC

**Table 2:** Primers for qRT-PCR analysis.

Gene Name	Primer Sequence (5′~3′)
GAPDH (F)	AACTTTGGCATTGTGGAAGG
GAPDH (R)	CCCTGTTGCTGTAGCCGTAT
Pro-glucagon (F)	AATCTTGCCACCAGGGACTT
Pro-glucagon (R)	AGTGACTGGCACGAGATGTT
PCSK1 (F)	TGGTGATTACACAGACCAGCG
PCSK1 (R)	CTCCAAGGCCAGAGCAAAGA

**Table 3:** Comprehensive scoring table for antioxidant activity of isolated strains.

Lactobacilli	Aggregate Score	Bifidobacterium	Aggregate Score
LGG	−1.05	BB12	−0.22
L-3	−0.13	6-1	0.31
L-8	−0.24	11-1	0.09
L-9	0.29	B-53	0.54
L-21	0.23	B-84	0.01

## Data Availability

Data is contained within the article.
